# Molecular mechanisms of avian immunoglobulin gene diversification and prospect for industrial applications

**DOI:** 10.3389/fimmu.2024.1453833

**Published:** 2024-09-13

**Authors:** Hidetaka Seo, Kouji Hirota, Kunihiro Ohta

**Affiliations:** ^1^ Department of Life Sciences, Graduate School of Arts and Sciences, The University of Tokyo, Tokyo, Japan; ^2^ Department of Chemistry, Graduate School of Science, Tokyo Metropolitan University, Tokyo, Japan

**Keywords:** gene conversion, somatic hypermutation, homologous recombination, DNA damage tolerance, monoclonal antibody, therapeutic antibody

## Abstract

Poultry immunoglobulin genes undergo diversification through homologous recombination (HR) and somatic hypermutation (SHM). Most animals share a similar system in immunoglobulin diversification, with the rare exception that human and murine immunoglobulin genes diversify through V(D)J recombination. Poultry possesses only one functional variable gene for each immunoglobulin heavy (HC) and light chains (LC), with clusters of non-productive pseudogenes upstream. During the B cell development, the functional variable gene is overwritten by sequences from the pseudo-variable genes via a process known as gene conversion (GC), a kind of HR. Point mutations caused in the functional variable gene also contribute to immunoglobulin diversification. This review discusses the latest findings on the molecular mechanisms of antibody gene diversification in poultry, using chickens as a model. Additionally, it will outline how these basic research findings have recently been applied especially in the medical field.

## 1 Introduction

The diversification mechanism of chicken immunoglobulin genes by GC was first identified in the 1980s through analysis of B cells derived from chicken bursa, which is an organ where hematopoiesis occurs. In human and murine immune system, it is well-known that immunoglobulin genes are diversified by V(D)J recombination, a site-specific recombination catalyzed by RAG 1/2 complex, which recognizes the signal sequence of V(D)J recombination (1). However, chicken immunoglobulin genes are predominantly diversified through HR (2–4).

**Figure 1:**
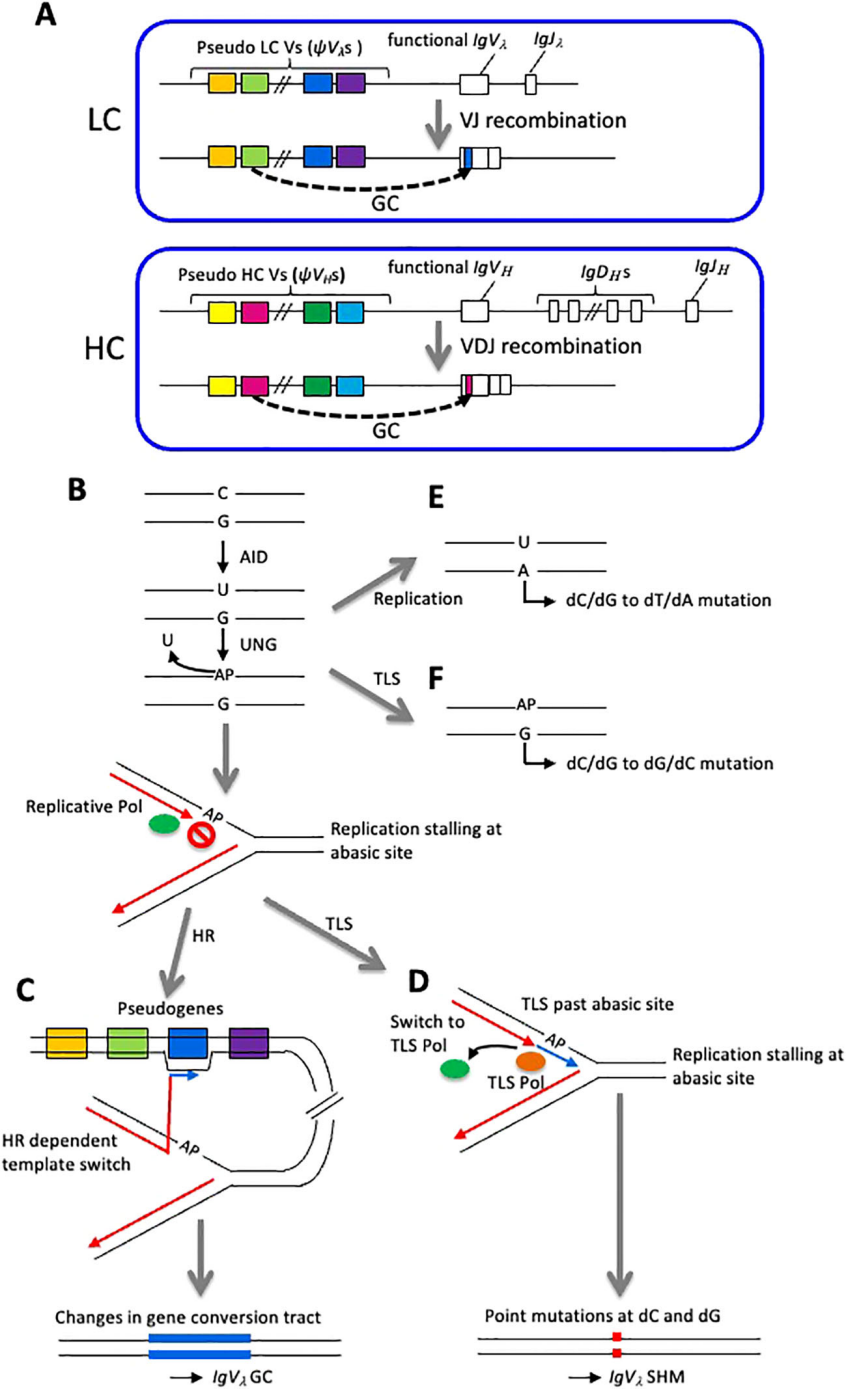
Chicken immunoglobulin locus and the mechanism of GC and SHM. **(A)** Genomic structure of chicken immunoglobulin LC and HC loci. **(B-F)** Schematically represented *IgV_λ_* diversification mechanism in DT40 cell line. **(B)** Deamination of cytidine followed by the action by UNG generates abasic site (AP) and replicative DNA polymerase stalls at AP site. **(C, D)** Template is switched to one of the *ψV_λ_* s, resulting HR mediated GC **(C)** or TLS-mediated SHM **(D)** events. **(E)** DNA replication assigns A to U generating dC/dG to dT/dA mutation. **(F)** TLS assigns G to abasic site, generating dC/dG to dG/dC mutation.

The structure of chicken immunoglobulin locus is described in **Figure 1A**. For the λ LC (chicken has only λ chain), there is only one functional variable (*IgV_λ_*) and one junctional (*IgJ_λ_*) gene, with 25 pseudogenes (*ψV_λ_* s) located upstream (2). Although these pseudogenes show homology to the functional *IgV_λ_*, none of them are productive. For example, they lack promoter and complete signal sequences for VJ recombination, and some pseudogenes have truncated 5’ or 3’ ends (2). During B cell development, VJ recombination occurs only once, contributing little to the diversification of LC genes since there are single functional *IgV_λ_* and *IgJ_λ_* gene each. However, GC occurs between the functional *IgV_λ_* and *ψV_λ_* s. In this process, partial sequences of pseudogenes are ‘copied’ and ‘pasted’ onto the functional *IgV_λ_* by HR. Interestingly, GC is a unidirectional event where *IgV_λ_* sequences are altered, but pseudogene sequences remain unchanged. Regarding the HC locus, it also contains only one functional V gene (*IgV_H_*) and J gene (*IgJ_H_*), but there are multiple (reported to be 16) diversity (*IgD_H_*) genes most of which are highly homologous with each other (3, 5). Similar to the LC, there is a cluster of pseudo V genes (*ψV_H_* s) upstream of functional *IgV_H_*, showing homology to the functional *IgV_H_* genes. The exact number of *ψV_H_* s is still not clear, with currently more than 80 HC pseudogene sequences from Red Junglefowl uploaded to the IMGT database (https://www.imgt.org). After VDJ recombination, GC also occurs between functional *IgV_H_* and pseudogenes, similar to LC. During B cell development, multiple rounds of GC generate primary diversity of immunoglobulin HC and LC loci. Diversification of immunoglobulin genes by GC is not limited to avian immune system; rabbits, cattle, swine, and horses also use GC to generate their immunoglobulin repertoire (6).

Numerous significant studies have been conducted to clarify the mechanisms of avian immunoglobulin diversification described above. Additionally, the elucidation of the molecular mechanisms of avian immunoglobulin diversification has not only deepened our understanding of immune system evolution but also holds promise for the biotechnological sector. These mechanisms can be harnessed in technologies for molecular evolution to acquire proteins with enhanced activities. For example, platform technologies have been developed for the *in vitro* generation and optimization of antigen-specific clones. Beyond their use as reagents, antibodies are also utilized as diagnostic and therapeutic tools. This underscores the significance of basic studies on avian immunoglobulin diversification.

## 2 Mechanism of *IgV* diversification

### 2.1 DT40 cell line

DT40 is a B cell line derived from the bursa of a chicken infected with avian leukosis virus (7). Interestingly, it was discovered that GC occurs continuously in the functional *IgV_λ_* of DT40 cells, albeit at a frequency much lower than *in vivo*. Further studies have also revealed that the frequency of targeted integration in this cell line is extremely high (8). The targeted integration rate is generally more than 50%, but varies depending on the targeting constructs or chromatin structure of the recipient locus. Leveraging these features, DT40 has been utilized as a tool for reverse genetics in various fields, including DNA repair, immunology, and chromosome structure. Notably, it has been instrumental in elucidating the mechanisms of GC and SHM at the immunoglobulin locus. Another distinctive feature of DT40 cells is their ability to express both membrane-bound and secreted types of IgM through alternative splicing. Moreover, DT40 cells exhibit exceptionally rapid growth as cultured cells, with a doubling time of about 8 hours, which facilitates various experiments.

### 2.2 Molecular mechanism of *IgV* diversification

The mechanism of *IgV_λ_* diversification has been extensively analyzed in chicken DT40 cells for understanding molecular mechanisms of DNA damage tolerance (DDT) system comprised of HR and translesion synthesis (TLS) (9). The *IgV_λ_* diversification is initiated by DNA replication fork arrest at the DNA damage on the template strand induced by activation-induced deaminase (AID) (10). AID induces the deamination of cytosine, thereby changing this base to uracil, which is removed by uracil-DNA glycosylase (UNG), an enzyme acting in the base excision repair (BER) pathway and becomes an abasic site (DNA damage without base) (11) (**Figure 1B**). In DNA replication, replicative polymerases δ and ϵ possessing extraordinarily high accuracy are believed not to be able to replicate across such abasic sites (12), and this DNA replication stalling triggers replication bypass either by HR-mediated template switching (leading to GC) or mutagenic TLS (leading to SHM) (**Figures 1B−D**). These *IgV_λ_* diversification events are most likely the consequence of the replication bypass across abasic sites, rather than the replication through dU as evidenced by the fact that most mutations are dC/dG to dG/dC transversions and loss of UNG instead leads to replication over the dU, resulting in dC/dG to dT/dA transitions (13) (**Figures 1E, F**). GC occurs between the *IgV*_λ_ locus and 25 copies of upstream-*ψV_λ_* segments carrying ~10% mismatch and thereby diversifies the *IgV*_λ_ gene (10, 14, 15) (**Figure 1C**). Meanwhile, mutagenic TLS induces hypermutations at C/G nucleotides to diversify the *IgV* gene (14, 16) (**Figure 1D**). Using DT40 cell line, many studies have assessed the contribution of factors to HR-mediated template switch and TLS via analyzing *IgV_λ_* diversification mechanisms. This chapter aims to discuss the latest findings on the DDT mechanisms involved in the *IgV_λ_* diversification in DT40 cells.

In the *IgV* gene diversification, GC is mediated through HR, as evidenced by the fact that deletion of all upstream *ψV_λ_* segments (homologous template for GC) completely abolishes GC and augments SHM (17). This observation also demonstrates that both GC and SHM are initiated from a common DNA lesion mediated by AID. This view is further supported by the observation that GC is also critically reduced but SHM is stimulated by the loss of Rad51 paralogs such as XRCC2/3, BRCA1, or BRCA2, all of which play critical roles in the recruitment of the Rad51 on DNA strands to activate HR (18–20). By sharped contrast, the loss of Rad54 or FANCD2, factors related to strand exchange in HR (21, 22), reduces GC without affecting the rate of SHM, suggesting that these factors contribute to GC after the strand exchange step in HR when the commitment towards GC has been made (23, 24). The polymerases involved in TLS might be used in the replication process of GC, since the concurrent loss of polymerase η, ν, and θ completely abolishes GC with a slight reduction of SHM (10). Conversely, several mutant cell lines show augmented GC. The loss of ASCIZ, a factor involved in the DNA base damage response (25), results in a marked increase in the rate of GC. These results suggest that this factor represses GC but induces BER of the abasic site (25). The loss of PolD4, the fourth subunit of polymerase δ, also increases the rate of GC. Because the concurrent loss of both PolD4 and ASCIZ has additive effects on GC and results in the drastic augmentation (~10 times increase) of GC rate, these two factors might play roles independently in the suppression of GC (26). The mechanism of how these factors repress GC has not been clarified.

The bypass replication by error-prone TLS polymerases causes non-templated SHM at the abacic site induced by the sequential action of AID and UNG (14, 16) (**Figure 1B**). Studies in budding yeast revealed that ubiquitination of PCNA at lysine 164 induces recruitment of TLS polymerases such as Polη to the sites of perturbed replication on the damaged template (27). The role of PCNA ubiquitination in *IgV* diversification was examined in *ψV_λ_* ^-^ DT40 cells in which all the upstream *ψV_λ_* genes are removed and GC events are diminished (17). The *PCNA-K164R/ψV_λ_* ^-^ cells showed impaired PCNA ubiquitination and exhibited hypersensitivity to a wide variety of DNA-damaging agents. Moreover, SHM at *IgV_λ_* gene was critically reduced in this cell line. These results indicate a critical role of PCNA ubiquitination on the cellular tolerance to DNA damaging agents as well as *IgV_λ_* gene SHM probably through inducing TLS. Rad18, an E3-ligase involved in the PCNA ubiquitination identified in yeast is conserved in eukaryotic cells (28, 29), and the deletion of the homologous gene for Rad18 in DT40 results in the augmented cellular sensitivity to DNA damaging agents and reduced *IgV* gene SHM (30). More importantly, *RAD18* ^-/-^/*PCNA-K164R* double mutant cells essentially show the same phenotype to *PCNA-K164R* mutant cells (30), indicating an epistatic relationship between *PCNA-K164* and *RAD18* ^-/-^. These results indicate that Rad18-mediated PCNA ubiquitination is required for TLS-mediated SHM events and other E3-ligase(s) serve as a backup for Rad18. *RAD18* ^-/-^/*PCNA-K164R* cells showed a reduction of dG:dC to dC:dG transversion, and a similar shift of mutation spectrum was also observed in *REV1* ^-/-^ cells (30). Moreover, the deoxytransferase activity of REV1 is required for *IgV_λ_* gene SHM but is dispensable for the cellular tolerance to DNA damaging agents (31). Taken together, these results suggest that the transferase activity of REV1 incorporates dC opposite to the abasic site under the regulation of PCNA ubiquitination by Rad18. *POLη* ^-/-^/*POLζ* ^-/-^ cells also exhibited reduced dG:dC to dC:dG mutation and augmented dG:dC to dA:dT mutation and totally this cell line showed a similar SHM rate compared with wild-type cells (16), suggesting that the absence of both Polη and Polζ is compensated by the action of other polymerase(s) possessing activity to incorporate dA opposite to abasic site such as Polδ (32). This view was supported by the observations that the loss of PolD3 (the third subunit of Polδ) reduces SHM but increases GC, and the loss of PolD3 in *POLη* ^-/-^/*POLζ* ^-/-^ has a lethal effect (14, 33). Moreover, both the SHM deficiency of *POLD3^-/-^* cells and lethality of *POLD3^-/-^*/*POLη* ^-/-^/*POLζ* ^-/-^ cells were rescued by the inactivation of proofreading exonuclease activity of Polδ (33). These results indicate that Polδ can contribute to SHM in parallel to Polη −Polζ axis, where PolD3 suppresses exonuclease-mediated excision of incorporated nucleotide opposite to the abasic site, thereby promoting SHM. The hypothesis that ‘Polδ can contribute to TLS-mediated SHM’ is totally unexpected, since the conventional dogma is that Polδ cannot bypass the damaged template and arrests. Other TLS polymerases, such as Polν and Polθ might also contribute to the promotion of SHM, since the inactivation of Polν and Polθ in *POLη* ^-/-^ cells significantly reduces SHM (10).

### 2.3 Regulation of GC by chromosome structure

It has also become evident that chromosomal structure plays a regulatory role on GC in immunoglobulin loci. Relaxed chromatin is known to be associated with high levels of histone acetylation and increased transcriptional activity (34). Similarly, processes such as chromatin remodeling and histone hyperacetylation occur prior to the initiation of meiotic recombination, which is a form of HR (35–37). It is suggested that GC at chicken immunoglobulin loci is regulated in a comparable manner. In fact, treating DT40 cells with trichostatin A (TSA), a histone deacetylase (HDAC) inhibitor, significantly enhances GC frequency, accompanied by increased histone acetylation status in the functional *IgVs* (15). Moreover, knocking out the HDAC2 gene in DT40 cells also increases GC frequency (38, 39). These results suggest that treatments with TSA or HDAC2 knockout relax the chromosomal structure of the immunoglobulin locus, likely enhancing the accessibility of DNA to various related factors mentioned above. Additionally, modifying DT40 cells to enable the binding of heterochromatin factor HP1—a factor necessary for heterochromatin formation—to their pseudogene region resulted in decreased histone acetylation levels nearby, which reduced GC frequency and increased SHM frequency. This reduction is probably attributable to the suppression of the pseudogene region by HP1, which in turn makes this region less efficiently utilized as a template for GC (40). This suggests that the chromosomal structure of not only the functional *IgV_λ_* but also the pseudogene region, which serves as a donor, is crucial for effective GC. DNA methylation is also one of the key mechanisms of epigenetic regulation (41). DT40 cells knocked out of TET3 (a member of Ten-eleven translocation family deoxygenase), which facilitates DNA demethylation show a marked reduction in GC activity at Ig locus (42). Depletion of TET3 in DT40 cells leads to hypermethylation in some pseudogenes, suggesting that TET3 is involved in the maintaining of their hypomethylation, thereby enabling these pseudogenes to serve as templates for GC.

## 3 Industrial application of GC and SHM using avian cells

### 3.1 Application of GC in avian cells

**Figure 2:**
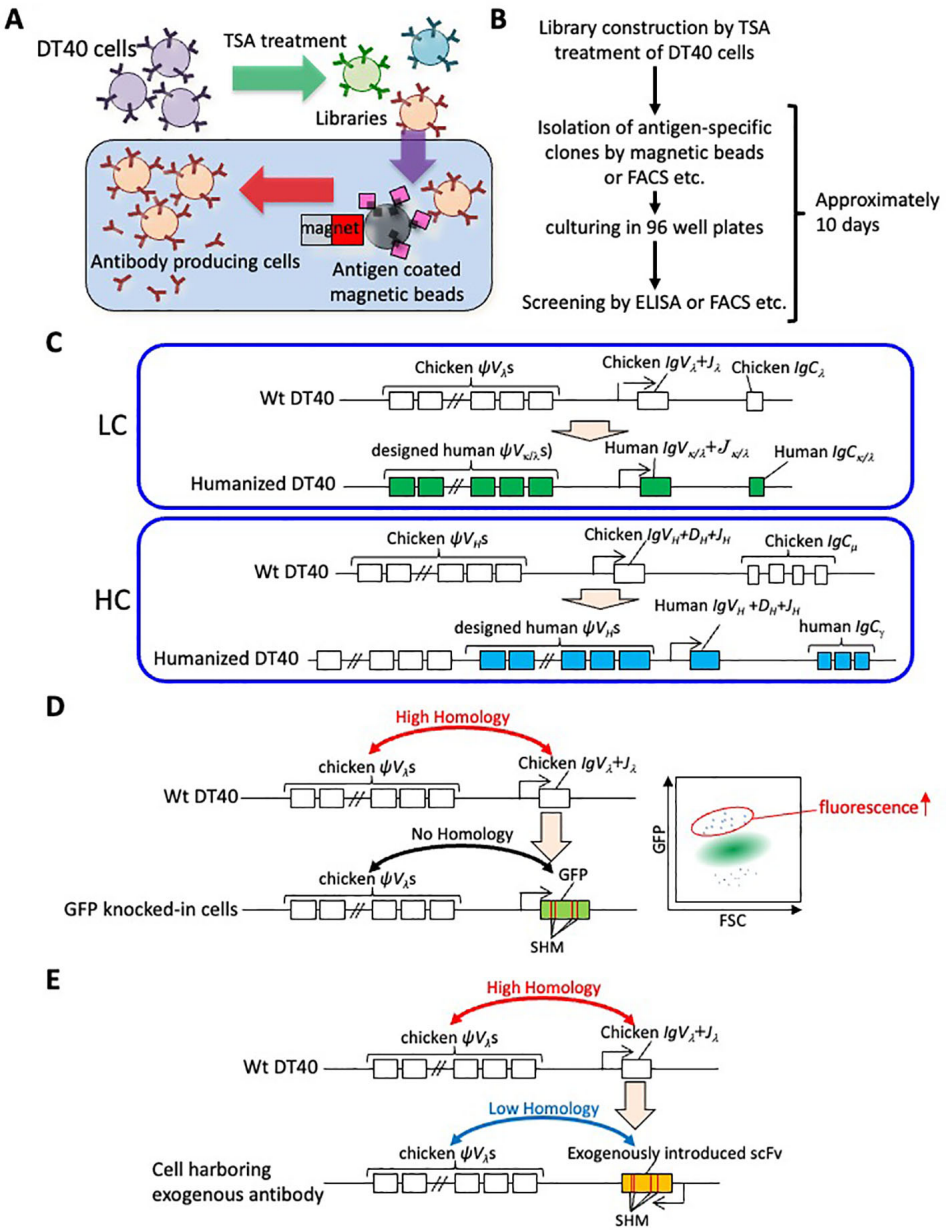
Application of GC and SHM using DT40 cells. **(A)** Principle of the ADLib system. DT40 cells are cultured with TSA to generate diversified cell-based mAb libraries. Antigen-specific clones can be isolated by, for example, antigen-coated magnetic beads. The isolated cells are expanded, and the antigen-specific monoclonal antibodies are recovered in culture supernatants. **(B)** Flowchart of the process for ADLib selection. The selection can be conducted using various methods, including antigen-conjugated magnetic beads and fluorescent cell sorting (FACS), among others. Validation of the antibodies can be performed using a range of methods including enzyme-linked immunosorbent assay (ELISA), FACS and other suitable methods. **(C)** Replacement and insertion of chicken immunoglobulin LC (upper) components (open rectangles) into human counterparts (green rectangles). The endogenous chicken pseudogenes are replaced with designed pseudogenes. Replacement of the chicken immunoglobulin HC (lower) components (open rectangles) with their human counterparts (blue rectangles). Designed pseudogenes are inserted downstream of the chicken pseudogene cluster. **(D)** Molecular evolution of GFP using DT40 cells. The GFP gene is introduced into immunoglobulin LC locus of DT40 cells and SHM accumulates (left panel). The diversified cells are analyzed by FACS assessing forward scatter (FSC) and GFP fluorescence. Cells with mutant GFP showing enhanced fluorescence are isolated. **(E)** Introduction of scFv gene to chicken immunoglobulin LC locus. Since scFv does not show homology to chicken *ψV_λ_* s, SHM occurred at introduced scFv gene.

As outlined above, significant progress has been made over the past 30 years in elucidating the molecular mechanisms of GC. Concurrently, there is an increasing trend in applying these basic research findings industrially, particularly concerning immunoglobulin gene rearrangement mechanisms in poultry. Antibody generation is a prime field where studies on GC have been effectively applied. Various *in vitro* antibody-generation methods, including phage and yeast display, have been developed to address the challenge of generating antibodies against evolutionarily conserved antigens, which often exhibit low immunogenicity (43, 44). As a novel *in vitro* method based on DT40 cells, a technology was developed that utilizes the enhancement of GC by TSA treatment for the antigen-specific antibody generation. Chicken IgM-presenting cell-based libraries are constructed by treating DT40 cells with TSA, then antigen-specific antibody-producing cells can be isolated using methods such as antigen-conjugated magnetic beads (**Figures 2A, B**). The rapid proliferation of DT40 cells and their capability to secrete IgM enable this autonomously diversifying library (ADLib) selection system to examine the antigen-specificities using culture supernatants or IgMs presented on the cell surface within approximately 10 days after selection (15, 45–47). To date, multiple antibodies with biologically functional activities obtained through the ADLib system have been reported (48–50), and several ADLib-derived antibodies have been approved as diagnostic drugs (LUMIPULSE G25-OH Vitamin D, LUMIPULSE Presto Aldosterone, and LUMIPULSE Presto iTACT Tacrolimus, from Fujirebio Inc.) (51). Besides the ADLib system, several laboratories have reported methods for the *de novo* generation of antigen-specific chicken IgM using DT40 cells (52–54).

The use of antibodies in the pharmaceutical field is expanding. Especially as therapeutics, the first antibody drug, an anti-CD3 antibody, was approved by the FDA in 1986 (55), and since then, therapeutic antibodies have been developed for various diseases, including cancer, autoimmune disorders and infectious diseases (56, 57). When monoclonal antibodies derived from non-human animals are administered to humans, they can induce immunogenicity, thus necessitating a process known as humanization (58). Humanization involves altering the amino acid sequences of non-human animal derived antibodies to increase their similarity to human antibodies (58), a laborious process that can sometimes alter the affinities or specificities of the antibodies. Since the antibodies produced by DT40 cells are chicken IgMs, antibodies obtained using the original ADLib system require humanization. To address this challenge, DT40 cells have been engineered where chicken immunoglobulin gene exons are replaced with human counterparts, and designed human variable gene sequences are inserted upstream as pseudogenes, constructing a cell-based library to isolate human IgG1 antibodies (human ADLib system) (**Figure 2C**) (59). The human ADLib system retains the simplicity and rapidity of the original ADLib system while enabling the production of human antibodies. In terms of human antibody generation, a method utilizing transgenic chickens has also been developed (60). Before this technology, transgenic animals including mice, rats, and rabbits were used to generate human monoclonal antibodies. However, as these animals are mammals which are phylogenetically related to humans, the range of candidate epitopes against human proteins is limited due to immunological tolerance. The advantage of chicken-based methods is the evolutionary distance between humans and chickens, which potentially offers a broader range of epitope candidates. In these transgenic chickens, the functional *IgV*s of both HC and LC are replaced with human counterparts, and designed human pseudogenes are inserted upstream of these functional *IgV*s. While the endogenous chicken LC constant region is replaced with that of humans, the HC constant region remains unaltered, allowing for immune responses against antigens in chicken. This transgenic chicken (OmniChicken) has successfully been used to generate human antibodies against human antigens, facilitated by robust immune responses. However, it should be noted that in both of the ADLib system and OmniChicken, SHM also occurs in addition to GC in the *IgV*s. Since GC and SHM share their mechanisms, they can occur simultaneously.

### 3.2 Application of SHM in avian cells

In addition to GC, technologies were developed for inducing SHM in DT40 cells to facilitate rapid molecular evolution. By utilizing the results that the DT40 cells knocked-out of XRCC2 accumulate SHM in immunoglobulin loci, a technology was developed that generates antigen-specific antibodies and maturates the affinity of them (61). They accumulated SHM in functional *IgV*s of the clones with no specificity to the antigens, and then sorted the clones with increased affinities to antigens. The iterative culturing and sorting process generated clones that show high affinity to the antigens. Other lab also reported the affinity maturation using DT40 cells hemizygously knocked-out of XRCC3 (62). Also, there have been successful instances where the GFP gene was introduced into the immunoglobulin LC locus of DT40 cells, and proteins with enhanced fluorescence intensity were obtained after iterative diversification followed by sorting process (**Figure 2D**) (63). In these experiments, the inserted GFP genes are diversified by SHM due to their lack of homology to chicken *ψV_λ_*. Among the cells harboring mutated GFP genes, some exhibited higher fluorescence intensity. Sorting these cells resulted in the acquisition of novel fluorescence proteins with fluorescence intensities up to as much as threefold higher than that of the parental GFP. This result, along with the finding from other lab (64), shows that non-antibody proteins can be evolved using the diversification mechanism of avian cells.

Besides, the method was developed for the affinity maturation of the antibodies in single chain fragment variable (scFv) format, in which V genes of HC and LC are linked as a single chain peptide (65). The scFv format is frequently used in technologies such as phage and yeast display, where the use of microorganisms, which are not well-suited for expressing full length antibodies, necessitates an alternative approach for full length antibody expression (43, 44). The scFv genes were knocked-in into the immunoglobulin LC locus of DT40 (**Figure 2E**). Since the introduced scFv antibodies are human or murine antibodies, their nucleotide sequences are less homologous to those of chicken pseudogenes, inducing SHM in scFv genes. The clones with improved affinities are sorted by staining with fluorescence labelled antigens followed by cell sorting. Additionally, a method has been developed for the fast-track affinity maturation of full-length antibodies, which involves introducing exogenous antibody’s V genes such as those from mouse hybridomas, into the *IgV_λ_* and *IgV_H_* of DT40 cells and inducing SHM (the ADLib KI-AMP system) (66). In the case of recombinant antibodies in the format such as scFv or Fab etc, further genetic engineering is required to reformat the isolated affinity-matured clones to IgG molecules. However, since the ADLib KI-AMP method facilitates the expression of complete LC and HC proteins, it directly yields the affinity-matured full-length IgGs. Consequently, the obtained antibodies can be easily used for immunoassays. If the human or humanized V genes are used for the ADLib KI-AMP, it can be the candidate of a therapeutic antibody.

## 4 Discussion

The studies on the unique mechanisms of GC and SHM in poultry immunoglobulin genes have significantly advanced our understanding of immunoglobulin diversification and its complex molecular basis. Utilizing DT40 chicken cell line, researchers have uncovered critical insights into these processes, illuminating broader genomic metabolism including DNA recombination and repair. Moreover, the basic studies on immunoglobulin gene diversification in poultry have now reached a stage where it is being applied in industrial and medical contexts. Future research is expected to delve deeper into the molecular intricacies of GC and SHM, potentially uncovering new ways to leverage these processes for broader biomedical applications.
